# The Dysregulation of the Integrated Stress Response in Leukemic Stem Cells as a Marker of Treatment Sensitivity in Acute Myeloid Leukemia

**DOI:** 10.3390/genes17080954

**Published:** 2026-08-14

**Authors:** Giorgia Benedetta Dutti, Katia Mangialardi, Simona Rasola, Ludovico Sebastio, Francesco Tarantini, Cosimo Cumbo, Luisa Anelli, Antonella Zagaria, Nicoletta Coccaro, Angela Minervini, Giuseppina Tota, Immacolata Redavid, Maria Rosa Conserva, Pellegrino Musto, Francesco Albano

**Affiliations:** 1 Hematology and Stem Cell Transplantation Unit, Department of Precision and Regenerative Medicine and Ionian Area (DiMePRe-J), University of Bari “Aldo Moro”, P.zza G. Cesare, 11, 70124 Bari, Italy; g.dutti@studenti.uniba.it (G.B.D.); k.mangialardi@studenti.uniba.it (K.M.); s.rasola@studenti.uniba.it (S.R.); l.sebastio2@studenti.uniba.it (L.S.); cosimo.cumbo@uniba.it (C.C.); luisa.anelli@uniba.it (L.A.); antonella.zagaria@policlinico.ba.it (A.Z.); giuseppina.tota@uniba.it (G.T.); immacolata.redavid@uniba.it (I.R.); maria.conserva1@uniba.it (M.R.C.); pellegrino.musto@uniba.it (P.M.); 2 Hematology and Stem Cell Transplantation Unit, Azienda Ospedaliero Universitaria Consorziale Policlinico, 70124 Bari, Italy; francesco.tarantini@policlinico.ba.it (F.T.); nicoletta.coccaro@policlinico.ba.it (N.C.); angela.minervini@policlinico.ba.it (A.M.)

**Keywords:** acute myeloid leukemia, integrated stress response, ISR, activating transcription factor 4, ATF4

## Abstract

Acute myeloid leukemia (AML) persistence is sustained by leukemic stem cells (LSCs) that survive metabolic deprivation, oxidative stress, hypoxia, proteotoxic burden, and therapeutic pressure. The integrated stress response (ISR) has emerged as a central adaptive network in this process. Through phosphorylation of a subunit of eukaryotic initiation factor 2 (eIF2α) and selective translation of activating transcription factor 4 (ATF4), the ISR coordinates stress-responsive transcriptional programs that may either preserve cellular fitness or promote apoptotic commitment, depending on the intensity, duration, and biological context of activation. In AML, ATF4 occupies a critical position at the interface between stemness, metabolic adaptation, redox control, ferroptosis resistance, and treatment response. In primitive leukemic compartments, ISR–ATF4 signaling appears to support stress tolerance, amino acid metabolism, serine biosynthesis, autophagy, and leukemic persistence. At the same time, pharmacologic or sustained ISR activation may lower the apoptotic threshold by inducing pro-apoptotic mediators such as CHOP, PUMA, and NOXA, thereby modulating MCL-1 dependency and enhancing sensitivity to venetoclax-based strategies. Conversely, adaptive ISR signaling may promote resistance through mechanisms such as ATP-binding cassette subfamily B member 1 (ABCB1) enhancer activation and mitochondrial stress tolerance. This duality creates a therapeutic paradox: ISR–ATF4 signaling may need to be inhibited in adaptive, resistance-promoting states but amplified in apoptosis-permissive contexts. This review discusses the biological and therapeutic relevance of ISR–ATF4 dysregulation in AML and highlights the need for biomarkers capable of distinguishing adaptive ATF4 dependency from inducible apoptotic vulnerability.

## 1. Introduction

Acute myeloid leukemia (AML) is a hematologic malignancy characterized by marked genetic, epigenetic, and clinical heterogeneity. Its defining feature is the clonal expansion of immature myeloid progenitors with impaired differentiation, leading to their accumulation in the bone marrow and peripheral blood [1]. Despite advances in molecularly targeted therapies and refinement of chemotherapy-based regimens, AML management remains limited by frequent relapse and the emergence of drug resistance [2,3]. A central reason for disease persistence is that a fraction of leukemic cells can survive conditions that would normally compromise cellular viability. Leukemic stem cells (LSCs), in particular, are highly adapted to metabolic deprivation, oxidative stress, proteotoxic burden, hypoxia, and therapeutic pressure, thereby sustaining disease propagation and relapse [4,5,6]. Understanding how these cells negotiate stress is therefore central to AML pathogenesis and treatment resistance. Accordingly, recent research has moved beyond the analysis of individual driver mutations toward the study of adaptive cellular states that allow LSCs to survive within an intrinsically hostile bone marrow microenvironment [4].

In this context, the integrated stress response (ISR) has emerged as a key stress-adaptive network. The ISR integrates diverse cellular insults through four kinases—protein kinase R-like endoplasmic reticulum kinase (PERK), protein kinase R (PKR), heme-regulated inhibitor kinase (HRI), and general control nonderepressible 2 (GCN2)—which converge on phosphorylation of the α subunit of eukaryotic initiation factor 2 (eIF2α). Phosphorylated eIF2α binds and inhibits eukaryotic initiation factor 2B (eIF2B), reducing the regeneration of active eIF2–GTP and the availability of the eIF2–GTP–Met-tRNAi ternary complex required for translation initiation. As a result, translation of most cellular mRNAs is attenuated. Activating transcription factor 4 (ATF4) mRNA represents an important exception because its upstream open reading frames act as a stress-sensitive translational switch, allowing increased ATF4 protein synthesis when ternary-complex availability is reduced [7]. Through this translational reprogramming, cells transiently conserve energy, restore proteostasis, and adapt to nutrient or oxidative stress. However, the ISR is not a uniformly cytoprotective pathway. Its biological output is strongly context-dependent: when stress is moderate or transient, ISR activation promotes adaptation and survival; when stress is severe, prolonged, or pharmacologically amplified, the same signaling axis may shift toward apoptotic commitment [4,7]. Although the ISR overlaps with the unfolded protein response (UPR) through the PERK–eIF2α–ATF4 branch, the two programs should not be considered synonymous. The UPR also includes inositol-requiring enzyme 1 alpha (IRE1α)/X-box binding protein 1 (XBP1) and activating transcription factor 6 (ATF6) signaling, whereas the ISR integrates broader stress inputs through multiple eIF2α kinases. This distinction is relevant in AML because endoplasmic reticulum (ER) stress, mitochondrial stress, amino acid deprivation, inflammatory signaling, and proteotoxic burden may activate partially overlapping but biologically non-identical stress-response programs [7,8].

This functional duality is particularly relevant in hematopoiesis and leukemia. Normal hematopoietic stem cells (HSCs) rely on tightly regulated stress-response programs to preserve quiescence, self-renewal, and long-term regenerative capacity. LSCs appear to co-opt these programs, using ISR signaling to tolerate hostile microenvironmental conditions and therapeutic stress [4]. Within this framework, ATF4 is not merely a downstream effector of ISR activation but a context-dependent determinant of leukemic fitness. By coordinating amino acid metabolism, redox homeostasis, proteostasis, and apoptotic priming, ATF4 occupies a strategic position at the interface between stress adaptation and cell fate [9]. Under adaptive conditions, ATF4 supports survival by restoring metabolic and proteostatic equilibrium [4]. Conversely, when stress becomes unresolved or is therapeutically intensified, ATF4 can contribute to cell death through induction of pro-apoptotic mediators such as C/EBP homologous protein (CHOP), p53 upregulated modulator of apoptosis (PUMA), and NOXA9. In AML, this duality creates a therapeutic paradox. ISR–ATF4 signaling may promote drug resistance by enhancing cellular fitness under treatment-induced stress, but under different conditions it may lower the apoptotic threshold and increase treatment susceptibility [10,11]. The ISR–ATF4 axis may therefore function as a double-edged biological program: a mechanism of leukemic persistence in one context and a therapeutically exploitable vulnerability in another. This distinction is especially relevant for venetoclax-based regimens, in which myeloid cell leukemia-1 (MCL-1) dependency, NOXA induction, mitochondrial stress signaling, and apoptotic priming critically influence therapeutic response [9,10].

On this basis, this review critically examines how the ISR–ATF4 axis regulates three interconnected dimensions of AML biology: first, LSC maintenance and protection within the bone marrow niche; second, metabolic reprogramming, with particular emphasis on serine biosynthesis and ferroptosis control; and third, therapeutic resistance, including evasion from apoptosis induced by B-cell lymphoma-2 (BCL-2) inhibition and conventional chemotherapy. Defining when ATF4-mediated adaptation should be inhibited, and when stress should instead be intensified to trigger apoptotic collapse, represents a central challenge for translating ISR biology into therapeutic strategies for AML.

## 2. ATF4, Leukemic Stemness, and Hierarchical Stress Adaptation

ATF4 is the principal downstream effector of the ISR and one of its most informative functional readouts. Following phosphorylation of eIF2α (p-eIF2α), global cap-dependent translation is attenuated, whereas the translation of selected stress-responsive transcripts is preserved or enhanced. Among these transcripts, ATF4 is the best characterized. This translational switch enables stressed cells to rapidly remodel gene expression to restore metabolic balance, maintain redox homeostasis, and adapt to proteotoxic or nutrient-related stress [12]. ATF4 is a basic leucine zipper (bZIP) transcription factor whose expression is controlled at multiple levels, including transcription, translation, and post-translational stability. Under basal conditions, ATF4 protein levels remain low because its mRNA contains two upstream open reading frames (uORFs) in the 5′ untranslated region. When the eIF2–GTP–Met-tRNAi ternary complex is abundant, ribosomes translate the permissive uORF1 and rapidly reinitiate at the inhibitory uORF2, which overlaps the ATF4 coding sequence and prevents efficient ATF4 translation. Following eIF2α phosphorylation, reduced ternary-complex availability delays ribosomal reinitiation after uORF1. Ribosomes therefore bypass uORF2 and instead initiate translation at the ATF4 coding sequence, increasing ATF4 protein production despite the overall reduction in global protein synthesis [12,13]. Once induced, ATF4 forms heterodimers with distinct transcriptional partners, generating context-dependent transcriptional outputs. Interaction with factors such as C/EBPβ or C/EBPγ may support adaptive recovery programs [11], whereas cooperation with CHOP is more commonly associated with pro-apoptotic signaling [13]. ATF4 should therefore not be regarded as a unidimensional marker of cellular stress but as a context-dependent regulator of cell fate downstream of ISR activation. This distinction is important when assessing ISR activity experimentally. Although p-eIF2α is the initiating event of the pathway, it is often transient and does not necessarily demonstrate that the downstream transcriptional program is functionally engaged. By contrast, ATF4 induction indicates propagation of ISR signaling to the transcriptional level [14]. Nevertheless, ATF4 should not be interpreted in isolation: its biological meaning depends on the amplitude, duration, cellular context, and composition of the downstream target-gene program [14]. For this reason, assessment of ATF4 protein expression should ideally be integrated with transcriptional profiling of canonical target genes and functional readouts of stress adaptation or apoptosis. In this sense, ATF4 represents a functional surrogate of ISR output rather than merely a secondary downstream molecule.

Normal HSCs must preserve long-term self-renewal while adapting to fluctuating metabolic and microenvironmental conditions. To achieve this balance, they rely on stress-adaptive programs that allow them to withstand amino acid deprivation, oxidative stress, hypoxia, DNA damage, and endoplasmic reticulum stress without losing functional integrity. Within this framework, ISR signaling appears to play an important role in maintaining primitive hematopoietic compartments [4,14]. Components of the ISR are differentially regulated across the hematopoietic hierarchy. Human HSCs and multipotent progenitors display lower protein abundance of the α, β, and γ subunits of eIF2 than downstream progenitors. This finding does not imply reduced ISR activity; rather, lower eIF2 availability is associated with a translational state that favors selective ATF4 expression. Consistently, ATF4 expression and ATF4 target-gene activity are enriched in stem and early progenitor populations [4]. This pattern is reinforced by xenograft-based analyses showing higher ATF4 expression in CD34^+^CD38^−^ HSC/MPP fractions than in more differentiated progenitor populations, both in vitro and in vivo [4]. ATF4 levels decline progressively along hematopoietic differentiation, suggesting that ISR activity is not uniformly distributed across precursor compartments but is linked to cellular immaturity. Importantly, cells with higher ATF4 expression display greater engraftment potential, indicating that ISR activity is associated with functional stem cell properties and not only with generic cellular stress [4].

This biological framework is highly relevant to leukemia. LSCs share with normal HSCs the ability to persist under adverse conditions and survive metabolic, oxidative, and microenvironmental stress. In AML, ISR-related features appear to be enriched in more primitive leukemic compartments. Expression of eIF2 subunits and ATF4 target genes differs between CD34^+^CD38^−^ and CD34^+^CD38^+^ AML fractions, and ATF4 expression is generally higher in CD34^+^ than in CD34^−^ leukemic cells [4]. Beyond the CD34/CD38 immunophenotype, several additional molecules have been associated with stem-like properties in AML, although they should not be regarded as interchangeable or universal LSC markers. CXCR4 is a functionally relevant chemokine receptor that promotes leukemic cell survival, homing, and engraftment within the bone marrow microenvironment; experimental CXCR4 blockade reduced the homing and in vivo propagation of primary human AML cells with leukemia-initiating activity [15]. Pluripotency-associated molecules have also been investigated in AML. In one patient-based study, OCT4 and SOX2 were more highly expressed in leukemic than in normal hematopoietic cells, whereas OCT4, SOX2, and SSEA3 were enriched in CD34^+^CD38^−^ compared with CD34^+^CD38^+^ AML fractions [16]. However, these associations were not supported by functional leukemia-propagation assays and do not establish these molecules as universal LSC markers. More recently, FLT3 inhibition was shown to induce OCT4 and NANOG, whereas SOX2 was not comparably increased in FLT3-ITD AML models. Depletion of OCT4 or NANOG impaired leukemic cell growth and enhanced sensitivity to gilteritinib [17]. These findings support a context-dependent contribution of pluripotency-associated transcriptional programs to FLT3-ITD AML maintenance and TKI resistance, rather than their use as pan-AML LSC markers.

However, these findings should be interpreted within the biological heterogeneity of AML, since ATF4 dependency is unlikely to be uniform across all genetic, phenotypic, and differentiation states. Functionally, LSCs may exploit ISR signaling as an adaptive program that sustains survival under nutrient deprivation, hypoxia, and increased reactive oxygen species. ATF4 upregulation in response to nutrient limitation, primarily through GCN2, promotes the transcription of amino acid transporters and biosynthetic enzymes, allowing leukemic cells to compensate for extracellular nutrient scarcity [18]. This metabolic plasticity may provide LSCs with a competitive advantage, supporting disease persistence under environmental and pharmacological stress.

The role of ATF4 in leukemic stemness is further supported by emerging evidence that epigenetic and post-transcriptional mechanisms can sustain its expression in selected AML contexts. Recent studies have implicated RNA methylation, particularly m6A modification, in this process. IGF2BP3, an m6A reader enriched in LSC-like populations, binds methylated sites on ATF4 mRNA and stabilizes transcripts of ATF4 and serine biosynthesis genes, thereby sustaining metabolic programs required for AML proliferation and stemness. Loss of IGF2BP3 or disruption of the METTL14-dependent m6A machinery reduces ATF4 levels and impairs leukemic cell survival in experimental models [19]. Preliminary preclinical data also suggest that BCLAF1 may link RNA splicing to ATF4-dependent metabolic adaptation, although this evidence remains based on a non-peer-reviewed preprint and should therefore be interpreted cautiously [20].

**Figure 1:**
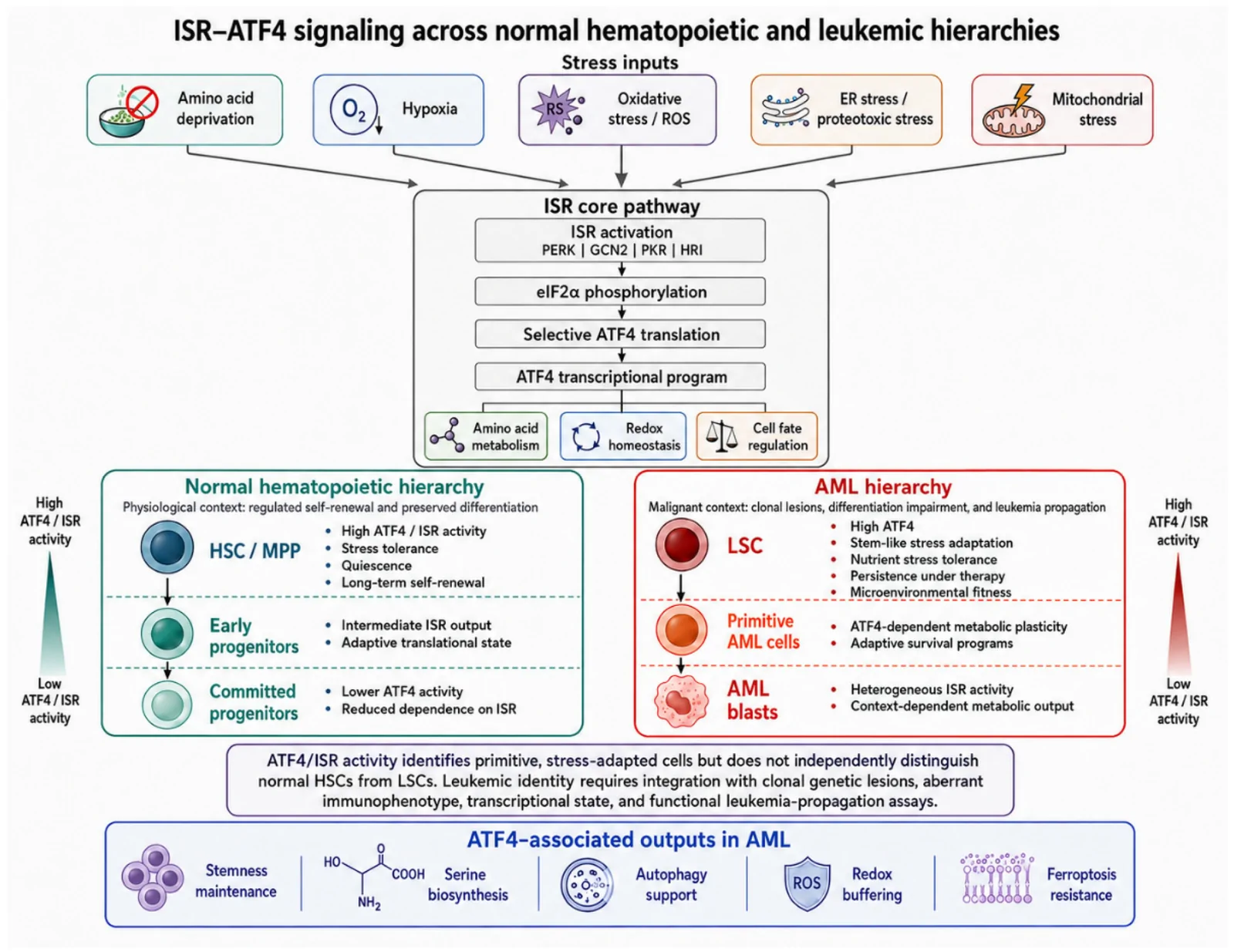
ISR–ATF4 signaling across normal hematopoietic and leukemic hierarchies. Diverse cellular stressors, including amino acid deprivation, oxidative stress, hypoxia, ER stress, and mitochondrial stress, converge on the ISR through PERK, GCN2, PKR, and HRI. This leads to eIF2α phosphorylation and selective translation of ATF4. In normal hematopoiesis, ISR–ATF4 activity is relatively enriched in primitive compartments and supports stress tolerance, quiescence, and long-term regenerative capacity. In AML, leukemic stem cells and primitive leukemic compartments appear to exploit the same program to sustain persistence, metabolic plasticity, redox buffering, and protection from ferroptotic stress. Importantly, high ISR–ATF4 activity is not specific to malignant hematopoiesis and cannot independently discriminate normal HSCs from LSCs. This distinction requires integration with leukemia-associated genetic lesions, aberrant immunophenotypic or transcriptional features, and functional evidence of leukemia-propagating capacity.

An additional layer of complexity is provided by the connection between ATF4 and the mechanistic target of rapamycin complex 1 (mTORC1) pathway. In specific leukemic models, ATF4 induces DEPTOR, an endogenous inhibitor of mTORC1, whose expression is further stabilized by MSI2. This ATF4–DEPTOR–KIF11 axis, in which KIF11 is a kinesin motor protein involved in mitotic spindle formation and cell-cycle progression, supports metabolic adaptation under nutrient stress and is required for efficient leukemic propagation in vivo [21]. These interactions between ISR–ATF4 activity, primitive hematopoietic compartments, leukemic stemness, and metabolic adaptation are summarized in Figure 1.

## 3. ATF4-Dependent Metabolic Reprogramming: From Serine Biosynthesis to Ferroptosis Resistance

If, in primitive leukemic compartments, ATF4 contributes to stress tolerance and stem-like persistence, in proliferating AML blasts it also functions as a metabolic coordinator [4,22]. AML cell survival depends on the ability to generate macromolecular precursors, sustain mitochondrial activity, and preserve redox equilibrium within a competitive and nutrient-limited bone marrow microenvironment. The metabolic relevance of ATF4 in AML can be organized around three interconnected outputs: amino acid biosynthesis, redox buffering, and resistance to regulated cell death. One of the best-characterized metabolic functions of ATF4 is the transcriptional regulation of the serine biosynthesis pathway (SSP), including phosphoglycerate dehydrogenase (PHGDH), phosphoserine aminotransferase 1 (PSAT1), and phosphoserine phosphatase (PSPH) [23]. In AML, serine supports one-carbon metabolism and de novo nucleotide synthesis, including purine and thymidylate synthesis. Recent studies indicate that the ATF4–SSP axis is hyperactivated in selected AML contexts, creating a dependency on endogenous serine production [24]. Dietary restriction of serine and glycine, or pharmacologic inhibition of PHGDH, can impair leukemic growth while relatively sparing normal hematopoietic cells in experimental models [19,24]. These findings suggest that the serine pathway is not simply a downstream metabolic consequence of leukemic growth but part of a regulated adaptive program linking RNA metabolism, ATF4 expression, and biosynthetic fitness.

Among genetically defined AML subsets, FMS-like tyrosine kinase 3 internal tandem duplication (FLT3-ITD) AML currently provides the most coherent evidence linking ATF4 to serine biosynthesis, autophagy, and stress-adaptive metabolic reprogramming. FLT3-ITD is one of the most frequent recurrent lesions in AML and defines a clinically relevant disease subset characterized by aggressive biology [1,25]. At the cellular level, oncogenic FLT3-ITD signaling also drives pronounced metabolic reprogramming [24]. Oncogenic FLT3-ITD signaling promotes serine synthesis and uptake through mTORC1–ATF4-dependent transcriptional regulation of de novo serine biosynthesis and neutral amino acid transport genes [24]. In this setting, ATF4 does not merely respond to exogenous stress but becomes integrated into oncogenic signaling circuits that support anabolic metabolism. Pharmacologic or genetic interference with serine biosynthesis, particularly through PHGDH inhibition, selectively impairs FLT3-ITD AML growth and sensitizes FLT3-ITD AML cells to cytarabine in experimental models [24].

ATF4 also intersects with autophagy in FLT3-ITD AML. Autophagy is a conserved lysosome-dependent catabolic process through which cells sequester and degrade damaged proteins, organelles, and other cytoplasmic components. The resulting macromolecular building blocks can then be recycled to preserve cellular homeostasis and sustain energy and nutrient availability during metabolic or environmental stress. In cancer, autophagy has a context-dependent role: although it may limit early tumorigenesis by preventing the accumulation of damaged cellular components, established malignant cells can exploit autophagic recycling to support proliferation, survival, and treatment resistance.

In FLT3-ITD AML, oncogenic signaling increases basal autophagic flux, and ATF4 has been identified as an essential mediator of this process [26]. Pharmacological or genetic inhibition of autophagy, as well as ATF4 depletion, reduced FLT3-ITD AML cell proliferation, decreased leukemic burden, and prolonged survival in xenograft models. Autophagy inhibition also overcame resistance associated with secondary FLT3 tyrosine kinase domain mutations in experimental models [26]. Notably, FLT3-ITD-dependent maintenance of ATF4 protein levels was reported to occur independently of eIF2α phosphorylation in this setting. Therefore, these findings establish an ATF4–autophagy dependency in FLT3-mutated AML, although they do not necessarily implicate canonical ISR activation as its upstream mechanism. Together, these observations illustrate how oncogenic FLT3 signaling may exploit ATF4-dependent autophagy to sustain metabolic adaptation, leukemic proliferation, and resistance to targeted therapy.

Beyond amino acid metabolism, ATF4 also contributes to redox control and ferroptosis resistance. Ferroptosis is an iron-dependent form of regulated cell death caused by the uncontrolled accumulation of lipid peroxides in cellular membranes [27]. One of the principal cellular defense systems against ferroptosis is the SLC7A11–glutathione–GPX4 axis. SLC7A11, also known as xCT, mediates cystine uptake in exchange for intracellular glutamate. Imported cystine is converted into cysteine and used for the synthesis of glutathione, which is required by glutathione peroxidase 4 (GPX4) to detoxify lipid hydroperoxides and prevent ferroptotic membrane damage. ATF4 can transcriptionally sustain SLC7A11 expression, thereby linking cellular stress signaling to antioxidant defense and ferroptosis suppression.

In AML, this pathway has been investigated in LSC-like cell lines and primary CD34+ AML cells. Combined HDAC3 inhibition and PPAR activation reduced ATF4-dependent transcriptional activity at the SLC7A11 locus, decreased SLC7A11 and GPX4 expression, depleted intracellular glutathione, and increased lipid peroxidation and ferroptotic cell death [28]. Notably, these effects were observed in primitive AML cells while relatively sparing normal CD34+ hematopoietic progenitors. These findings identify the ATF4–SLC7A11–glutathione–GPX4 pathway as a candidate redox-survival mechanism in LSC-like compartments, although its therapeutic relevance remains based on preclinical evidence.

A further level of regulation is provided by post-transcriptional RNA modifications. AlkB homolog 3 (ALKBH3) is an RNA demethylase that removes N1-methyladenosine (m1A) modifications from RNA, potentially influencing mRNA stability and translation. In KG-1 AML cells, ALKBH3 knockdown increased m1A modification of ATF4 mRNA and reduced ATF4 expression. This was accompanied by decreased expression of the ferroptosis-protective proteins SLC7A11, GPX4, and FTH1, together with increased intracellular ferrous iron, reactive oxygen species, lipid peroxidation, and expression of ferroptosis-associated markers such as acyl-CoA synthetase long-chain family member 4 (ACSL4), PTGS2, and TFR1 [29]. In xenograft experiments, ATF4 overexpression partially reversed the growth-inhibitory effects of ALKBH3 depletion, supporting a functional ALKBH3–m1A–ATF4 axis.

These findings suggest that ALKBH3 may preserve an ATF4-dependent anti-ferroptotic program in AML. However, the available evidence is derived predominantly from KG-1 cells, a xenograft model, and a limited number of clinical samples. Therefore, the relevance of this mechanism across genetically and phenotypically diverse AML subsets remains to be established. Moreover, this mechanism regulates ATF4 at the RNA level and should not necessarily be interpreted as evidence of canonical eIF2α-dependent ISR activation. Similarly, m6A-dependent mechanisms involving IGF2BP3 stabilize ATF4, PHGDH, and PSAT1 transcripts, sustaining serine production and supporting AML stemness and metabolic resilience [19]. Although these findings require broader validation across genetically diverse AML cohorts, they suggest that RNA modification pathways can tune ATF4-dependent metabolic adaptation and influence vulnerability to oxidative and ferroptotic stress. Rather than acting as a single master switch, ATF4 should therefore be viewed as one component of a broader stress-metabolic network that integrates oncogenic signaling, RNA regulation, amino acid availability, and redox state.

## 4. The ISR–ATF4 Axis as a Mediator of Dynamic Drug Resistance

**Figure 2:**
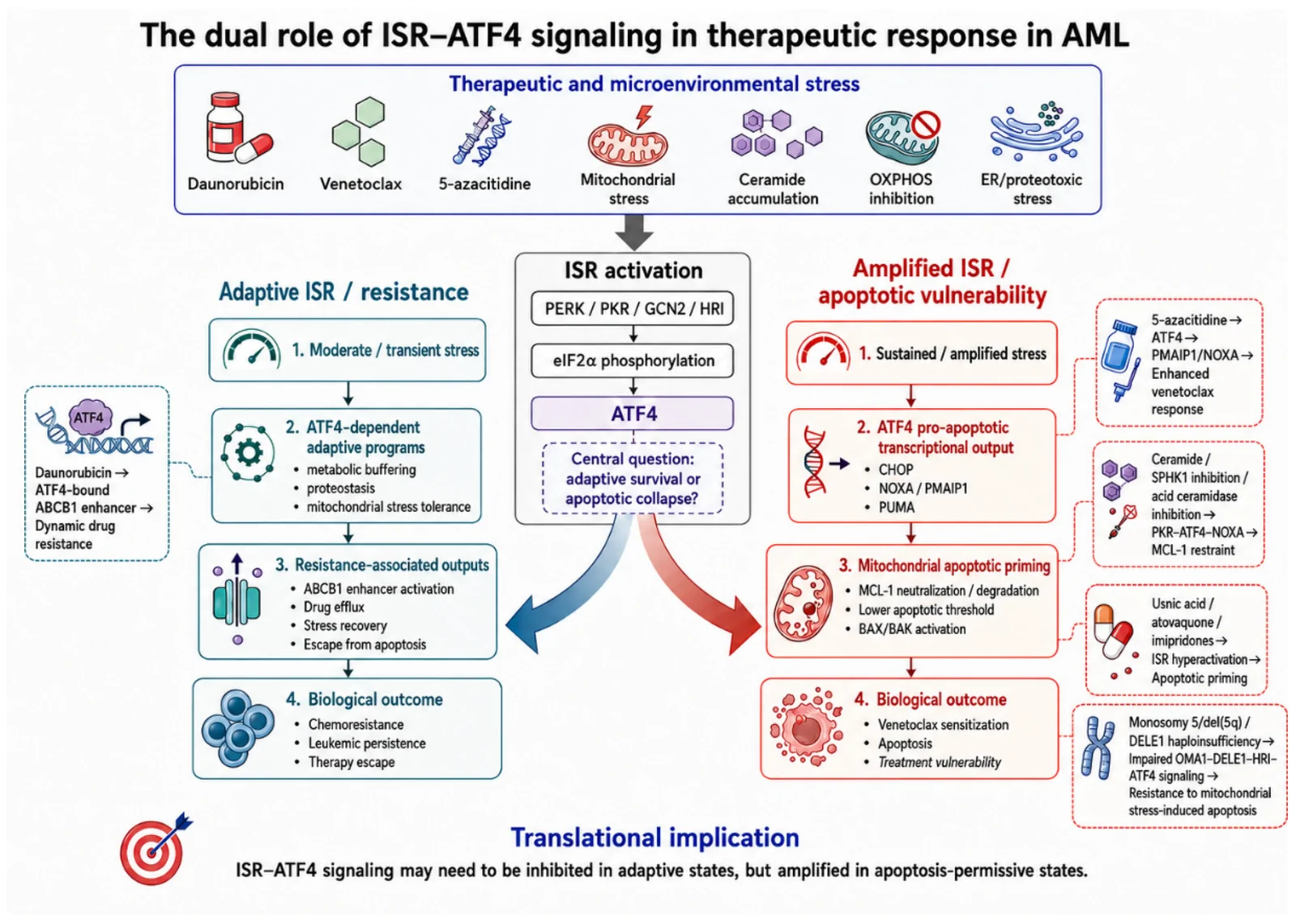
The dual role of ISR–ATF4 signaling in therapeutic response in AML. Therapeutic and microenvironmental stressors converge on ISR activation and ATF4 induction. When stress is moderate or transient, ATF4-dependent adaptive programs may support leukemic survival through metabolic buffering, mitochondrial stress tolerance, and enhancer-mediated chemoresistance, including ABCB1 activation. By contrast, sustained or pharmacologically amplified ISR signaling may promote CHOP-, NOXA-, and PUMA-associated apoptotic priming, neutralize MCL-1-dependent resistance, and enhance sensitivity to venetoclax-based or other pro-apoptotic therapies. The key translational challenge is to identify when ISR signaling should be inhibited and when it should instead be amplified to force leukemic collapse.

In AML, the ISR influences treatment response in a highly context-dependent manner. Moderate or transient ISR activation may enhance leukemic fitness under therapeutic pressure and contribute to drug resistance, whereas sustained or excessive signaling may promote apoptotic commitment [10,11]. This functional duality is particularly relevant in AML, where therapeutic efficacy often depends on the balance between adaptive stress tolerance and mitochondrial apoptotic priming. The introduction of venetoclax has transformed AML treatment, but primary and acquired resistance remain major clinical challenges. A common mechanism of resistance involves a shift in anti-apoptotic dependency from BCL-2 toward alternative proteins such as MCL-1 or BCL2A1. The ISR–ATF4 axis can modulate this process in opposite directions depending on the nature and intensity of the stress signal [10]. The context-dependent effects of ISR–ATF4 signaling on adaptive resistance and apoptotic vulnerability are schematically illustrated in Figure 2.

Mitochondrial stress can activate a dedicated branch of the ISR through the OMA1–DELE1–HRI pathway. OMA1, a protease located in the inner mitochondrial membrane, cleaves the mitochondrial protein DELE1 in response to organelle dysfunction. Cleaved DELE1 accumulates in the cytosol and activates HRI, leading to eIF2α phosphorylation and ATF4 induction. In AML, this pathway has been particularly characterized in cases with monosomy 5 or del(5q), because DELE1 is located within the commonly deleted region of chromosome 5q. In these leukemias, DELE1 haploinsufficiency reduces mitochondrial stress-induced ATF4 activation and protects AML cells from apoptosis. Thus, in this genetically defined context, an intact OMA1–DELE1–HRI–ATF4 relay contributes to mitochondrial stress-induced apoptotic vulnerability, whereas pathway attenuation promotes resistance [30].

Other forms of mitochondrial or metabolic stress can similarly shift ISR output toward apoptosis. Ceramide accumulation, achieved through inhibition of sphingosine kinase 1 or acid ceramidase, activates a PKR–eIF2α–ATF4 cascade that induces the BH3-only protein NOXA. NOXA neutralizes MCL-1 and, in several experimental contexts, promotes its degradation, thereby reducing a major mechanism of venetoclax resistance and restoring apoptotic sensitivity [10,31,32]. A similar logic applies to hypomethylating agent–venetoclax combinations. 5-azacitidine has been shown to activate ISR-associated transcriptional programs and promote ATF4-dependent induction of PMAIP1 (NOXA), thereby facilitating MCL-1 neutralization and enhancing venetoclax-induced apoptosis [10].

Another mechanism through which ISR signaling may favor treatment resistance involves transcriptional upregulation of drug-efflux machinery. A well-characterized example is ATP-binding cassette subfamily B member 1 (ABCB1), whose expression can be induced downstream of ATF4 activation [11]. Exposure to daunorubicin activates a stress-responsive enhancer at the ABCB1 locus, with ATF4 binding driving transcriptional activation. Importantly, this enhancer can remain in a primed state, enabling faster reactivation upon subsequent drug exposure and contributing to dynamic chemoresistance [11]. ATF4-dependent resistance is further reinforced by interactions with broader stress-response networks. JUN, a key regulator of the UPR in AML, is activated downstream of MEK/ERK signaling during endoplasmic reticulum stress and can enhance transcription of UPR effectors, including ATF4. Inhibition of JUN or its downstream targets reduces the ability of leukemic cells to cope with proteotoxic stress and promotes apoptosis [8].

This dual biology creates two complementary therapeutic strategies. First, the adaptive arm of the ISR can be inhibited to prevent leukemic cells from exploiting ATF4-dependent survival programs. ISRIB, an eIF2B activator, counteracts the effects of eIF2α phosphorylation and limits ATF4 induction. In preclinical models, ISRIB reduces stress-induced ABCB1 enhancer activation and sensitizes AML cells to daunorubicin [11]. Second, the apoptotic arm of the ISR can be deliberately hyperactivated. Imipridones such as ONC201, ONC212, and ONC213 induce atypical mitochondrial stress, leading to robust ATF4 activation and induction of pro-apoptotic mediators such as CHOP, PUMA, and NOXA. Through NOXA-mediated MCL-1 modulation, these agents can overcome venetoclax resistance and induce apoptosis even in p53-deficient settings [33,34,35]. Other pharmacologic approaches follow similar principles. Atovaquone suppresses oxidative phosphorylation while activating ATF4-dependent stress signaling, thereby targeting AML cells reliant on mitochondrial metabolism [36]. Usnic acid activates the HRI–eIF2α–ATF4 axis, induces NOXA, promotes MCL-1 downregulation, and sensitizes venetoclax-resistant AML cells [37].

The strength and translational maturity of the available evidence vary considerably across AML contexts. The most consistent genotype-specific data currently concern FLT3-ITD AML, in which ATF4 supports serine biosynthesis and autophagy-dependent leukemic fitness [24,26]. A second genetically defined context is monosomy 5/del(5q) AML, where DELE1 haploinsufficiency impairs the OMA1–DELE1–HRI–ATF4 relay and promotes resistance to mitochondrial stress-induced apoptosis [30]. More recent studies have identified additional genotype-associated ATF4 programs. In NUP98::DDX10 leukemia, NOL10 cooperates with the fusion protein to stabilize ATF4 mRNA and sustain serine biosynthesis, whereas CEBPA p30-mutant cells display altered AP-1 and ATF4 responses to inflammatory and endoplasmic reticulum stress [38,39]. These latter findings broaden the spectrum of genetically defined AML contexts associated with ATF4 dysregulation, although they do not yet establish uniform activation of the canonical ISR.

Despite compelling mechanistic evidence, the translational maturity of ISR-directed strategies remains heterogeneous. Most approaches discussed above—including serine biosynthesis inhibition, autophagy targeting, ceramide modulation, imipridones, atovaquone, and usnic acid—remain supported predominantly by cellular, xenograft, or patient-derived preclinical models. One notable example of clinical translation is the GSPT1 degrader CC-90009, whose activity involves activation of the GCN1–GCN2–ATF4 pathway and which entered early-phase clinical evaluation in AML in studies registered as NCT02848001 and NCT04336982 [18]. However, no ISR- or ATF4-based biomarker has yet been prospectively validated for treatment selection. The central translational challenge is therefore to determine when ISR inhibition is preferable to ISR hyperactivation. AML with high IGF2BP3 expression or FLT3-ITD may be particularly dependent on ATF4-driven metabolic adaptation [19,24]. Conversely, AML with monosomy 5 or del(5q), characterized by DELE1 haploinsufficiency, may have impaired mitochondrial ISR signaling and reduced responsiveness to ISR activators relying on the DELE1–HRI axis [30]. Ultimately, effective therapeutic exploitation of the ISR–ATF4 axis will require biomarkers capable of distinguishing adaptive ATF4 dependency from inducible apoptotic vulnerability. The main biological and therapeutic contexts in which ISR–ATF4 signaling has been implicated in AML are summarized in Table 1.

## 5. Future Directions and Open Questions

Although increasing evidence supports a relevant role for ISR signaling in AML biology and treatment response, several key questions remain unresolved (Table 1). A central challenge is that ISR output is unlikely to depend simply on whether the pathway is activated or inactive. Rather, its biological consequences are likely determined by the magnitude, duration, kinetics, and cellular context of activation. This distinction is particularly important in AML, where transient ISR engagement may support adaptation, whereas sustained or amplified signaling may promote apoptotic priming [4,10]. Future studies should also define which upstream eIF2α kinase drives ATF4 induction in each AML context since GCN2-, HRI-, PKR-, and PERK-driven ISR outputs may differ in kinetics, transcriptional consequences, and therapeutic exploitability. This issue is not merely taxonomic: amino acid deprivation, mitochondrial stress, ceramide accumulation, ER stress, and inflammatory or proteotoxic signals may all converge on ATF4 while producing distinct downstream programs [4,10,11,30,31,37].

A second major issue concerns biomarker development. ATF4 is a biologically informative readout of ISR activity, but its value as an in vivo biomarker in AML remains insufficiently defined. Longitudinal assessment of ATF4 expression at diagnosis, during treatment, and at relapse could clarify whether dynamic changes in ISR output correlate with stem-like features, treatment sensitivity, or emerging resistance. Future studies should move from single-marker ATF4 assessment toward integrated ISR signatures combining ATF4 expression, canonical target genes, upstream kinase activation, and functional apoptotic readouts. The mechanisms that terminate or buffer ISR activation also deserve greater attention. Growth arrest and DNA damage-inducible protein 34 (GADD34) and constitutive repressor of eIF2α phosphorylation (CReP)-containing phosphatase complexes regulate eIF2α dephosphorylation, whereas ISRIB functionally counteracts eIF2α phosphorylation by activating eIF2B. These regulatory nodes may determine whether AML cells resolve stress and survive or fail to restore translational homeostasis and undergo apoptosis. Therefore, therapeutic modulation of the ISR should be understood not only as pathway activation or inhibition but also as manipulation of signal duration, recovery kinetics, and translational reset [7,12,14]. Safety and preservation of normal hematopoiesis represent another critical translational issue. Because ISR activity contributes to the maintenance of normal primitive hematopoietic compartments, therapeutic modulation of the ISR–ATF4 axis will require careful assessment of hematopoietic toxicity. A key question is whether leukemic cells and normal HSCs differ sufficiently in ISR amplitude, kinetics, upstream kinase dependency, or apoptotic threshold to provide a clinically useful therapeutic window [4,7]. These considerations also raise important therapeutic questions. In hypomethylating agent–venetoclax regimens, it remains unclear whether sequential drug exposure, cumulative stress signaling, or treatment timing influences ISR-dependent apoptotic priming. More broadly, pharmacologic manipulation of ISR signaling deserves further investigation both as a strategy to enhance pro-apoptotic output and as a means of suppressing adaptive stress programs associated with resistance [10,11,14]. At present, however, these strategies remain hypothesis-generating and require validation in biologically relevant in vivo models and patient-derived systems. Taken together, the next phase of research should move beyond descriptive evidence of ISR activation and define when, where, and to what extent this pathway becomes functionally relevant in AML. Such an approach may clarify whether ISR signaling can serve not only as a mechanistic explanation for leukemic persistence but also as a framework for biomarker-driven therapeutic intervention. Ultimately, the translational promise of ISR biology in AML will depend on the ability to distinguish adaptive ATF4 dependency from inducible apoptotic vulnerability. Converting stress adaptation into treatment vulnerability may represent one of the most promising therapeutic implications of this pathway.

## Figures and Tables

**Table 1:** Functional contexts of ISR–ATF4 signaling in AML.

Context	ISR/ATF4 Input	Main Output	Biological Consequence	Therapeutic Implication	Key References
HSC/LSC hierarchy	Low eIF2 availability and high ATF4/ISR activity in primitive compartments	Stress tolerance and preservation of primitive cell function	Maintenance of normal HSC integrity and enrichment of stem-like leukemic states	Potential functional marker of primitive stress-adapted compartments; requires caution because normal HSCs also use ISR signaling	[4,7,14]
FLT3-ITD AML	Oncogenic FLT3-ITD signaling converges on mTORC1–ATF4 programs	Serine biosynthesis, neutral amino acid transport, and autophagy	Metabolic dependency and enhanced anabolic/stress-adaptive capacity	PHGDH inhibition, autophagy targeting, or metabolic combinations with chemotherapy	[24,26]
m6A/RNA-processing programs	IGF2BP3/METTL14-mediated stabilization of ATF4 and serine biosynthesis transcripts; preliminary BCLAF1–ATF4 splicing axis	Sustained ATF4 protein expression and amino acid biosynthetic programs	Support of AML stemness, proliferation, and metabolic resilience	Targeting RNA-modification or splicing-dependent metabolic dependencies; BCLAF1 data remain preliminary	[19,20]
Ferroptosis control	ATF4-driven SLC7A11/xCT, GPX4, and redox-protective programs; ALKBH3-mediated ATF4 upregulation	Cystine import, glutathione synthesis, and lipid peroxide buffering	Protection from ferroptotic injury and oxidative stress	xCT/GPX4/ferroptosis-inducing strategies, especially in LSC-like compartments	[27,28,29]
Ceramide/SPHK1 or acid ceramidase inhibition	Ceramide accumulation activates PKR–eIF2α–ATF4 signaling	NOXA induction and MCL-1 neutralization/degradation	Restoration of apoptotic sensitivity in venetoclax-resistant AML	ISR hyperactivation as a strategy to enhance BCL-2 inhibition	[10,31,32]
Daunorubicin exposure	ATF4-bound stress-responsive ABCB1 enhancer activated by chemotherapy-induced stress	ABCB1 upregulation and enhancer priming	Dynamic chemoresistance and drug efflux	ISRIB/eIF2B modulation or enhancer-directed strategies to limit adaptive resistance	[11]
Monosomy 5/del(5q) AML	DELE1 haploinsufficiency impairs OMA1–DELE1–HRI–ATF4 mitochondrial stress signaling	Reduced ATF4 activation after mitochondrial stress	Resistance to mitochondrial stress-induced apoptosis	Potential biomarker for poor responsiveness to ISR activators relying on the DELE1–HRI axis	[30]
Mitochondrial stress pharmacology	Imipridones, atovaquone, and usnic acid induce mitochondrial or HRI-linked ISR activation	ATF4/CHOP/NOXA induction, oxidative phosphorylation suppression, and MCL-1 modulation	Apoptotic priming and reversal of selected resistance states	Candidate combination strategies with venetoclax or chemotherapy; clinical validation remains limited	[33,34,35,36,37]

Summary of major biological and therapeutic contexts in which ISR–ATF4 signaling has been implicated in AML. The table emphasizes that ATF4-dependent outputs differ according to upstream stress input, leukemic cell state, and apoptotic threshold.

## Data Availability

No new data were created or analyzed in this study. Data sharing is not applicable to this article.

## Abbreviations

The following abbreviations are used in this manuscript:

AML	acute myeloid leukemia
LSCs	leukemic stem cells
ISR	integrated stress response
eIF2α	α subunit of eukaryotic initiation factor
ATF4	activating transcription factor 4
CHOP	C/EBP homologous protein
PUMA	p53 upregulated modulator of apoptosis
MCL-1	myeloid cell leukemia-1
ABCB1	ATP-binding cassette subfamily B member 1
PERK	protein kinase R-like endoplasmic reticulum kinase
PKR	protein kinase R
HRI	heme-regulated inhibitor kinase
GCN2	general control nonderepressible 2
eIF2B	eukaryotic initiation factor 2B
UPR	unfolded protein response
IRE1α	inositol-requiring enzyme 1 alpha
XBP1	X-box binding protein 1
ATF6	activating transcription factor 6
ER	endoplasmic reticulum
HSCs	hematopoietic stem cells
BCL-2	B-cell lymphoma-2
p-eIF2α	phosphorylation of eIF2α
bZIP	basic leucine zipper
uORFs	upstream open reading frames
C/EBP	CCAAT/enhancer-binding protein
HSC/MPP	hematopoietic stem cell/multipotent progenitor
CXCR4	C-X-C motif chemokine receptor 4
OCT4	octamer-binding transcription factor 4
SOX2	SRY-box transcription factor 2
SSEAR	stage-specific embryonic antigen 3
FLT3	FMS-like tyrosine kinase 3
NANOG	Nanog homeobox
FLT3-ITD	FLT3 internal tandem duplication
TKI	tyrosine kinase inhibitor
m6A	N6-methyladenosine
IGF2BP3	insulin-like growth factor 2 mRNA-binding protein 3
METTL14	methyltransferase-like 14
BCLAF1	BCL2-associated transcription factor 1
mTORC1	mechanistic target of rapamycin complex 1
DEPTOR	DEP domain-containing mTOR-interacting protein
MSI2	Musashi RNA-binding protein 2
KIF11	kinesin family member 11SSP
ROS	reactive oxygen species
SSP	serine biosynthesis pathway
PHGDH	phosphoglycerate dehydrogenase
PSAT1	phosphoserine aminotransferase 1
PSPH	phosphoserine phosphatase
SLC7A11	solute carrier family 7 member 11
GPX4	glutathione peroxidase 4
HDAC3	histone deacetylase 3
PPAR	peroxisome proliferator-activated receptor
ALKBH3	AlkB homolog 3
m1A	N1-methyladenosine
FTH1	ferritin heavy chain 1
ACSL4	acyl-CoA synthetase long-chain family member 4
PTGS2	prostaglandin-endoperoxide synthase 2
TFR1	transferrin receptor 1
BCL2A1	BCL2-related protein A1
OMA1	OMA1 zinc metallopeptidase
DELE1	DAP3-binding cell death enhancer 1
SPHK1	sphingosine kinase 1
BH3	BCL-2 homology 3
MEK	mitogen-activated protein kinase
ERK	extracellular signal-regulated kinase
ISRIB	integrated stress response inhibitor
NUP98::DDX10	nucleoporin 98–DEAD-box helicase 10 fusion
NOL10	nucleolar protein 10
CEBPA	CCAAT/enhancer-binding protein alphaAP-1
GSPT1	G1 to S phase transition 1
GCN1	general control nonderepressible 1
GADD34	growth arrest and DNA damage-inducible protein 34
CReP	constitutive repressor of eIF2α phosphorylation
